# On the Pathology and Treatment of Gonorrhœa and Spermatorrhœa

**Published:** 1887-09

**Authors:** 


**On the Pathology and Treatment of Gonorrhœa and Spermatorrhœa.** By J. L. Milton, Senior Surgeon to St. John's Hospital for Diseases of the Skin, London. Octavo, 484 pages. Illustrated. Price, bound in extra muslin, $4.00. New York: William Wood & Co.

This is a book which has the merit of thoroughness. It is not introduccd for a class-book, but rather for the use of the experienced practitioner. The aim of the author has been "to separate clearly what might be looked on as established from what was doubtful, and not merely to prove every assertion, but to place it on such a basis that it could not be disproved." In this he has succeeded sufficiently well to make a book which ought to be in the hands of every practitioner who sees anything of venereal disease.

